# Burden and social context of caregivers of children and adolescents with Charcot-Marie-Tooth disease

**DOI:** 10.1055/s-0046-1829046

**Published:** 2026-10-02

**Authors:** Caroline Soares de Paula, Juliana Cardoso, Wilson Marques Junior, Cyntia Rogean de Jesus Alves de Baptista, Ana Claudia Mattiello-Sverzut

**Affiliations:** 1 Universidade de São Paulo, Faculdade de Medicina de Ribeirão Preto, Departamento de Ciências da Saúde, Ribeirão Preto SP, Brazil.; 2 Universidade de São Paulo, Faculdade de Medicina de Ribeirão Preto, Departamento de Neurociências e Ciências do Comportamento, Ribeirão Preto SP, Brazil.

**Keywords:** Hereditary Sensory and Motor Neuropathy, Caregiver Burden, Educational Status, Socioeconomic Factors

## Abstract

**Background:**

In pediatric Charcot-Marie-Tooth (CMT) disease, caregivers play an important role in coordinating care and promoting child well-being. Therefore, understanding caregiver burden and social context is key for treatment planning.

**Objective:**

To investigate caregiver burden, family income, and level of schooling among caregivers of children and adolescents with CMT disease, and to examine correlations between caregiver burden, patient age, clinical impairment, and caregiver age.

**Methods:**

A cross-sectional study was conducted with 58 patients (aged 3–18 years) and their primary caregivers. Caregiver profile and burden were obtained through interview. The Caregiver Burden Scale (CBS) assessed burden, and the Brazilian version of the Charcot-Marie-Tooth Pediatric Scale (CMTPedS-Br) assessed patient's clinical impairment.

**Results:**

The median age of children and adolescents was of 13.0 (11.0–15.0) years, and that of caregivers was of 41.0 (36.5–46.0) years. Median scores were 22.5 (18.0–27.0) for CMTPedS-Br and 31.5 (25.2–44.8) for CBS. Most caregivers were mothers (85.2%), did not present a CMT diagnosis (59.6%), earned R$ 2,200 to 4,400 per month (42.6%), and completed high school (33.3%). Burden did not differ by income or level of schooling and showed a weak positive correlation ( *p* = 0.17) with caregiver age only.

**Conclusion:**

Caregivers of children and adolescents with CMT disease and moderate impairment may experience burden. Although socioeconomic factors were not decisive in explaining differences in caregiver burden, their variability highlights the need to consider them in care planning. Caregiver burden is a complex construct that may involve additional factors not included in the present study. Overall, findings support multidisciplinary, caregiver-centered interventions in pediatric CMT disease care.

## INTRODUCTION

Charcot-Marie-Tooth (CMT) disease represents the most common hereditary neuropathy. 1 2 3 In general, signs and symptoms begin in childhood and are characterized by distal muscle weakness and distal sensory deficits affecting both the upper and lower limbs. 4 5 6

As a rare condition with no cure, 7 treatment is largely supportive and includes physiotherapy, occupational therapy, orthotic devices, and home-based rehabilitation programs. 8 9 10 11 12 Proper medical follow-up and rehabilitation therapies aim to optimize functional performance, prevent secondary complications, and support participation and long-term independence in daily life. 13 14 However, evidence from high-quality studies is not always translated into practice, often because implementation aspects, such as real-world feasibility, are overlooked. 15 16

In this regard, attention to the caregiver can be a key factor. In childhood and adolescence, disease management is usually entrusted to a primary caregiver, 17 18 who plays a crucial role in supporting daily functioning, coordinating care, and promoting the child's wellbeing. Research on neuromuscular diseases such as Duchenne muscular dystrophy (DMD) and spinal muscular atrophy (SMA) shows that these caregivers often experience high levels of burden, associated with factors such as a child's high level of dependency and financial burden. 19 20 21 However, there is a lack of research on this topic in the context of CMT disease, in which case the caregiver burden may be even more complex, as caregivers themselves can also be affected by the condition, further increasing both demands and vulnerability.

Caregiver wellbeing is increasingly recognized as an essential component of family-centered care, given its potential impact on treatment adherence, rehabilitation outcomes, and overall quality of life. Moreover, understanding the social context of the target population is vital to implement clinical trial findings and design interventions that are both effective and feasible in real-life settings. 15 16

Therefore, the present study aimed to investigate burden, family income, and the level of schooling of caregivers of children and adolescents with CMT disease. It further examined whether clinical impairment and age of the patient, as well as caregiver age, were correlated with caregiver burden, and whether this burden differs according to the caregiver's social context.

## METHODS

### Study design and participants

The present cross-sectional study included 58 children and adolescents of both sexes diagnosed with CMT, aged between 3 and 18 years. Patients and caregivers were recruited from the Children's CMT Outpatient Clinic at a Rehabilitation Center.

The current study was approved by the ethics committee (No.: 50692021.0.0000.5440). Before data collection began, all patients and caregivers signed an informed consent form.

### Data collection

The genetic diagnosis of the children and adolescents was retrieved from electronic medical records. Children and adolescents with a clinical diagnosis and a family history of CMT, but without a confirmed genetic diagnosis, were classified as “genetically undiagnosed.”

Clinical impairment was assessed using the Charcot-Marie-Tooth Pediatric Scale 22 (CMTPedS-Br), which has been translated and validated for Brazilian Portuguese. 23 The CMTPedS-Br comprises 11 items covering hand function, distal muscle strength of lower limbs, distal sensation, balance, and gait. Following the assessment, the results for each item were entered into the CMTPedS calculator, available at https://www.clinicaloutcomemeasures.org/ . The calculator assigns a score from 0 to 4 points to each item; these are summed to yield a total score of up to 44 points. Total scores of 0– to 14 indicate mild impairment, 15 to 29, moderate impairment, and 30 to 44, severe impairment. 5

Caregiver information was collected in two stages. In the first stage, an in-person interview was conducted with the primary caregiver to obtain information on the caregiver-patient relationship, monthly family income, schooling level, and caregiver burden. In the second stage, a telephone interview was conducted with the primary caregiver to collect self-reported data on caregiver age and CMT disease diagnosis. In this stage, seven caregivers did not respond to the call; therefore, data on caregiver age and diagnosis were not obtained for the entire sample. The information on the diagnosis of the caregiver was confirmed in the electronic medical records.

Caregiver burden was assessed using the Caregiver Burden Scale (CBS), 24 which has been translated and validated for Brazilian Portuguese. 25 The CBS comprises 22 items covering general strain, isolation, disappointment, emotional involvement, and environment. Each item is scored on a 4-point scale, and the total score (maximum of 88 points) is calculated by summing all items. Scores range from 0 to 88, according to which 0 represents no burden and 88 represents the maximum possible burden score.

### Statistical analysis

For statistical analysis, the RStudio software version 2026.01.0 + 392 (Posit Software, PBC) was used. The distribution of the data was verified graphically. Continuous variables were presented as median and interquartile range (1 ^st^ quartile [Q1] to 3 ^rd^ quartile [Q3]). Categorical variables were presented as frequency (%).

The Kruskal-Wallis test verified whether caregiver burden differed when income and level of schooling were considered ( *p* < 0.05). Schooling data were grouped into no schooling (illiterate and incomplete primary school), primary school (complete primary school and incomplete secondary school), secondary school (complete secondary school and incomplete higher education), and higher education (complete higher education or postgraduate degrees). 26

The correlations between caregiver burden and patient clinical impairment and between caregiver burden and patient age were calculated using the Spearman's correlation coefficient (r). The correlation between caregiver burden and caregiver age was calculated using the Pearson's correlation coefficient (r). Values of r = 1.00 indicated perfect correlation; r = 0.90 to 0.80, strong; r = 0.70 to 0.50, moderate; r = 0.40 to 0.10, weak; and r = 0.09 to 0.00, no correlation. 27 Correlations were considered statistically significant when *p* < 0.05.

## RESULTS

The anthropometric characteristics and diagnoses of the patients ( *n* = 58) are presented in Table 1 . On the CMTPedS-Br, the sample had a median score of 22.5 (18.0–27.0) points, which corresponds to moderate clinical impairment. Five (8.6%) patients used a wheelchair for short-distance mobility.

**Table 1:** Sample characteristics

Children and adolescents ( *n* = 58)	Median (Q1–Q3)
Age (years)	13.0 (11.0–15.0)
Height (cm)	158.5 (144.0–167.2)
Weight (kg)	51.6 (34.3–64.4)
Sex	**n (%)**
Female	24 (41.4)
Male	34 (58.6)
Subtype	**n (%)**
CMT1A	40 (69.0)
CMT1B	5 (8.6)
CMT2A	5 (8.6)
CMTX1	2 (3.5)
CMT1D	1 (1.7)
CMT2C	1 (1.7)
CMT2K	1 (1.7)
CMT2N	1 (1.7)
Genetically undiagnosed	2 (3.5)

Abbreviations: CMT, Charcot-Marie-Tooth disease; n, number of cases; Q1, first quartile; Q3, third quartile.

The caregiver sample comprised 54 participants, including four caregivers who were relatives of two children each. Regarding the relation to the children, most caregivers were mothers (85.2%), followed by fathers (9.3%), and grandparents (5.6%). Considering the missing data from the phone interviews, the median age of caregivers ( *n* = 47) was 41.0 (36.5–46.0) years, and 40.4% had a diagnosis of CMT disease. Charcot-Marie-Tooth disease diagnosis information could be confirmed in the electronic medical records for all caregivers except two.

For caregiver burden, a total of 58 CBS responses were analyzed, as caregivers of two children provided separate responses for each child. The median CBS score was 31.5 (25.2–44.8). Figure 1 presents the distribution of CBS scores according to the presence or absence of CMT in caregivers.

**Figure 1:**
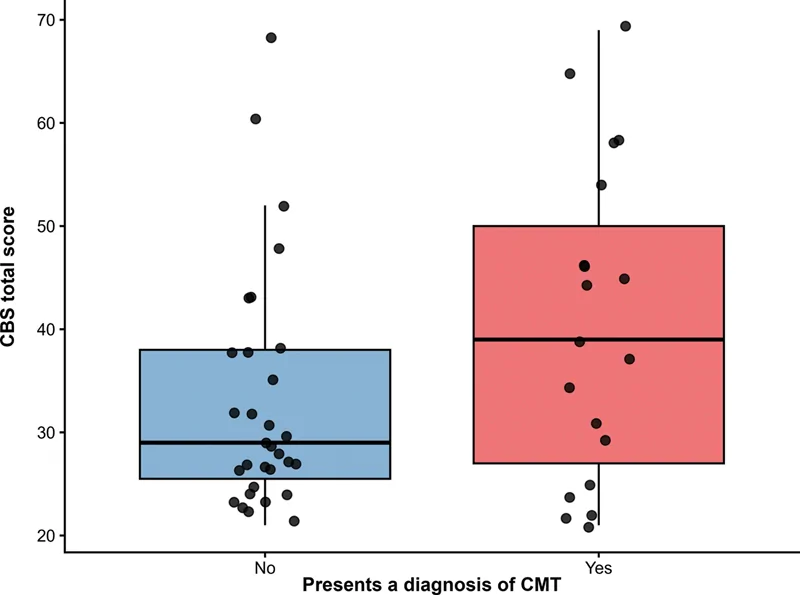
Distribution of the caregivers ( *n* = 47) with and without Charcot-Marie-Tooth disease diagnosis.

For the income data, only 4 caregivers had an income above R$ 11,000, and were included in the income group of R$ 4,401 to R$ 11,000. Among the caregivers ( *n* = 54), 42.6% had a monthly income between R$ 2,200 and R$ 4,400, and 33.3% had completed higher education.

The total score of the CBS scale and the CMTPedS-Br obtained in each income and schooling subgroup are presented in Table 2 . When comparing caregiver burden across different income and schooling levels, no evidence of differences was found (schooling: Kruskal-Wallis χ ^2^ (3) = 2.28; *p* = 0.51; income: Kruskal-Wallis χ ^2^ (2) = 2.86; *p* = 0.24).

**Table 2:** Median values of the total score of the CMTPedS-Br and CBS for each level of income and schooling level

	CBS score ( *n* = 58) Median (Q1-Q3)	CMTPedS-Br score ( *n* = 58) Median (Q1-Q3)
**Income** (n/%)		
Up to R$ 2,200 (18/31.0)	38.0 (27.5–44.7)	22.0 (18.2–28.5)
R$ 2,201–4, 400 (25/43.1)	25.0 (24.0–37.0)	23.0 (18.0–27.0)
R$ 4,401–11,000 (15 /25.9)	35.0 (26.0–49.0)	20.0 (17.5–24.0)
**Level of Schooling** (n/%)		
No schooling (10/17.0)	41.5 (26.7–53.2)	23.0 (18.2–28.2)
Primary school (10/17.0)	29.0 (25.5–52.5)	22.5 (19.2–27.0)
Secondary school (19/33.0)	28.5 (24.0–38.5)	18.0 (13.0–25.5)
Higher education (19/33.0)	34.0 (28.5–40.5)	24.0 (20.0–29.5)

Abbreviations: CBS, Caregiver Burden Scale, CMTPedS-Br, Charcot-Marie-Tooth Pediatric Scale; n, number of cases; Q1, first quartile; Q3, third quartile.

Patient clinical impairment and patient age were not correlated with the level of caregiver burden (r = -0.00, *p* = 0.97; r = 0.02, *p* = 0.86, respectively) ( Figure 2A,B ). Caregiver age presented a positive and weak correlation with caregiver burden (r = 0.20, *p* = 0.17) ( Figure 2C ).

**Figure 2:**
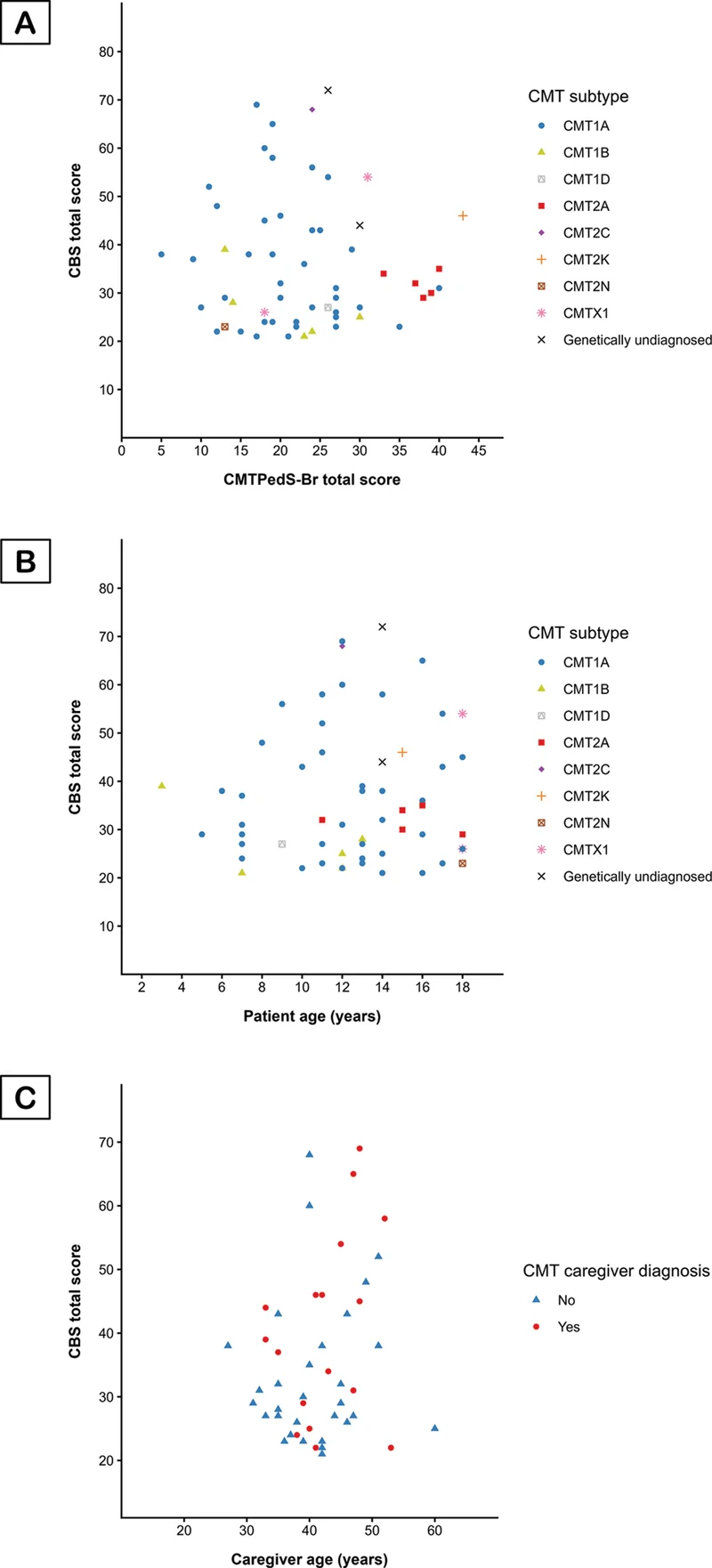
Scatter plots showing the correlations between caregiver burden and ( **A** ) clinical impairment, ( **B** ) patient age, and ( **C** ) caregiver age.

## DISCUSSION

In the current study, caregivers presented a median score of 31.5 points on the CBS (maximum score: 88 points), which suggests that caregivers of children and adolescents with CMT disease in this sample experience some level of burden. These findings may reflect the clinical characteristics of the sample, with ∼ 70% of patients diagnosed with CMT1A and presenting moderate clinical impairment. Although CMT disease is genetically and clinically heterogeneous, 1 4 CMT1A, the most common subtype, 4 6 typically presents with moderate impairment and slow disease progression. 5 6 As a result, individuals with CMT1A may sustain greater independence in daily activities, which could help explain the median score of caregiver burden observed in the present study.

Based on these characteristics, a positive correlation between clinical impairment and caregiver burden would be expected. However, the distribution of burden scores was heterogeneous across levels of clinical impairment. Caregivers of patients with moderate impairment showed wide variability, while those caring for patients with severe impairment (three wheelchair users) did not necessarily report higher burden. These findings suggest that, although clinical impairment plays an important role in patient independence, it may not be the main determinant of caregiver burden. A similar pattern was observed for patient age. It could be expected that caregivers of younger children, who may be more dependent, or of older adolescents, who may present greater disease progression, 4 would report higher burden. However, higher CBS scores were observed at specific ages (12, 14, and 16 years), reinforcing the absence of correlation between patient age and caregiver burden.

Among the variables analyzed, caregiver age was the only one showing a weak, non-significant positive correlation with burden, suggesting that older caregivers may experience greater burden. Nevertheless, the heterogeneous distribution of CBS scores suggests that caregiver burden in this study is unlikely to be explained by a single factor, but rather reflects a complex, multifactorial construct. In addition to the variables investigated in this study, other aspects may contribute to this heterogeneity, such as availability of social support, accumulation of roles (e.g., caregiving combined with employment), access to psychological support, comorbidities and engagement in physical activity, all of which may modify stress. 28 29

Although socioeconomic factors such as family income and schooling are often associated with caregiver burden, 30 31 no differences in burden were observed in our sample when these factors were considered. Most caregivers reported a monthly family income between R$ 2,201 and R$ 4,400, corresponding to ∼1.5 to 3.0 minimum wages at the time of data collection. In CMT disease, there are currently no disease-modifying pharmacological treatments, and care is primarily based on rehabilitation and physical activity. 8 This contrasts with other neuromuscular conditions, such as SMA and DMD, which often require more complex and costly care. 30 32 33 Additionally, although rehabilitation services may represent a financial burden in some healthcare systems, 34 in Brazil these services are available free of charge through the public healthcare system, which may reduce the financial impact on families.

Regarding schooling level, 66.7% of caregivers had completed secondary or higher education, which may facilitate understanding of the disease and its management and potentially contribute to lower burden. In contrast, 33.3% had low levels of formal education or were not literate, which may represent a barrier to effective communication. These last findings reinforce the importance of providing clear, accessible, and tailored information to families. Effective communication may improve treatment adherence and support caregivers in managing the demands of the condition, 35 36 potentially reducing stress related to uncertainty or lack of information.

Another important aspect in CMT disease is that caregivers may themselves be affected by the disease or may be responsible for more than one affected family member. In our sample, caregivers without CMT disease tended to report lower CBS scores, whereas those with CMT disease showed greater variability in CBS scores. This suggests that having the disease may impact the caregiving experience and highlights the need for targeted support strategies for this group.

Finally, it is important to acknowledge the potential influence of social desirability bias, in which participants provide responses perceived as more socially acceptable rather than fully reflecting their experiences. 37 The CBS includes sensitive questions, such as feelings of frustration or the desire to withdraw from the caregiving situation, which may be difficult to report, particularly in the context of interaction with healthcare professionals. This may partially explain why caregivers of patients with higher levels of clinical impairment did not report higher burden scores.

The present study has some limitations. The relatively small sample size limited subgroup analyses across CMT disease subtypes. In addition, the sample was predominantly drawn from a single region and medical center, which may limit generalizability of the findings. Missing data from telephone interviews also prevented complete characterization of caregiver age and diagnosis, and the diagnosis of two caregivers could not be confirmed through medical records. Furthermore, information on private health insurance, psychological therapy, number of children, occupation, and physical activity was unavailable, although it could have provided a more comprehensive understanding of the caregiver profile. Despite these limitations, the current study contributes to the limited literature on caregiver burden in CMT disease, providing insights on the social context of these patients and the implications for caregiver burden. These findings may inform future research and support the development of targeted, multidisciplinary interventions to better support caregivers.

### Relevance for the clinical practice

Our findings highlight that there is a caregiver burden in CMT disease. Routine evaluation of caregiver burden, regardless of patient impairment level, is important and may help identify those at risk of increased stress, particularly older caregivers and those who may also be affected by the disease. In addition, interventions should extend beyond physical rehabilitation and include strategies to enhance support, promote caregiver well-being, and address role overload. The variability in schooling levels observed in this sample also highlights the need for clear, accessible, and tailored communication to improve understanding of disease management and support adherence to care.
